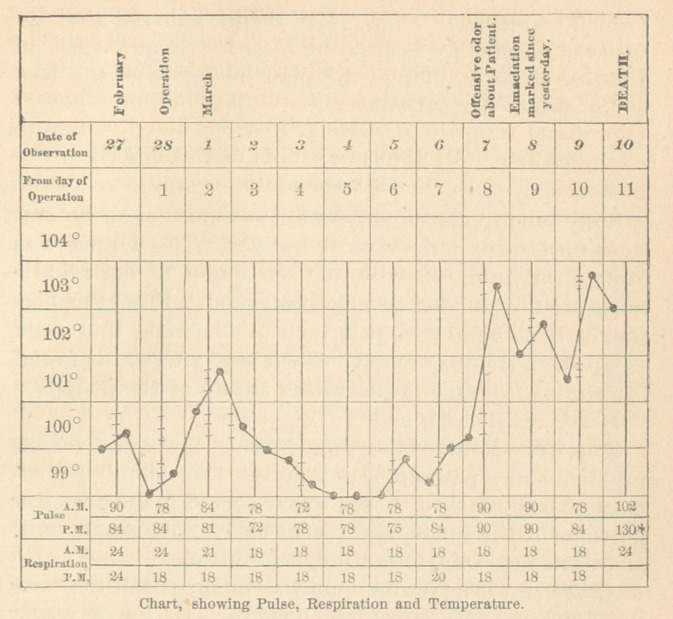# Hospitals

**Published:** 1875-01

**Authors:** 


### Hospitals.

### ST. LUKE'S HOSPITAL, CHICAGO.

*Colloid, Multilocular-Ovarian Tumor—Ovariotomy—Septicæmia—Death.*

(Under the care of JNO. E. OWENS, M.D.)

Mrs. C., aged 57 years; a thin, sallow woman, and of perfectly even temper; was admitted to the hospital February 24, of the present year. Seven months previous to admission, she discovered a tumor in the *left* iliac region. The tumor had increased in size, so that the patient presented the appearance of one advanced to the eighth month of pregnancy. Fluctuation was quite distinct, and the mass freely movable from side to side. Several cysts were made out. Mrs. C. was the mother of seven children, five of which are living—the last being thirteen years old. Menstruation ceased seven months before admission. The animal functions were regularly performed, the urine normal, and the uterus not enlarged. The general health had not very materially failed. The patient had never been tapped. Dr. De Laskie Miller was of the opinion that the comparatively rapid growth of the tumor suggested a malignant element in the case.

The tumor was removed February 28. The patient not being easily affected with æther, chloroform was substituted, until the production of anæsthesia, which was then maintained with æther. The incision began one and a half inches below the umbilicus, and extended towards the pubis four and a half inches. The abdominal walls were very thin, and but a few drops of blood appeared in the line of the incision. There were no adhesions. A large cyst, with very fragile walls, presented. During the manipulation of this, in order to introduce the trocar, the cyst wall ruptured, exhibiting almost colorless, jelly-like, colloid matter, remaining *in situ*, even after a tolerably free laceration of the cyst-wall. The patient having been turned on the left side, the colloid matter was scooped out. Other cysts were ruptured, and voided through the first one, till by gentle traction and by pressure the tumor was delivered. It was a degeneration of the *right* ovary, and attached by a well formed pedicle, which, together with the cysts, can usually be the most conveniently managed by turning the patient upon the side opposite to that from which the tumor grows. The vermiform appendix was greatly distended with colloid matter, and presented, upon its external aspect, minute cyst-like collections of the same. The peritoneal lining of the lower coils of intestine was inflamed, and resembled in condition, "granular lids." The pedicle having been secured by Atlee's clamp, the abdominal wound was closed by means of three pins extending through the peritoneal lining, and as many silk sutures penetrating the skin only. The lower end of the wound was left open for the sake of drainage. A wad of cotton batting and a flannel roller completed the dressing. The patient was put in a warm bed, in a well ventilated room, and a two-grain opium suppository placed in the rectum.

The tumor weighed sixteen pounds. The patient died of septicæmia, on the tenth day from the date of the operation.

During the first four days, the case progressed favorably. There was no pain, the patient slept well, without anodynes, and seemed perfectly undisturbed in every respect. There was an occasional regurgitation of the fluid contents of the stomach. This was effected, however, without effort, and without change of position. She took ice *ad libitum.* The diet consisted of milk toast in the morning and evening, and beef tea at intervals through the day and night. At the end of the third day the nurse noticed an offensive odor about the patient, when the bed clothing was disturbed. On the fifth day the wound having united, except at its lower end, and at that portion through which the pedicle passed, I removed one pin, a silk suture and the clamp. Upon the removal of the tent from the lower extremity of the wound, one or two drachms of blood-tinged pus escaped. The remains of the pedicle were black and offensive.

7th day. An occasional regurgitation—an offensive odor about the patient, arising from the sloughy remains of the pedicle. By the traction of the latter, at its point of attachment to the abdomen, the wound was drawn inwards, making quite a deep cup-like cavity. This was kept open with carbolized lint; ordered an injection of warm castor oil (one ounce), which in an hour effected a copious and painless evacuation. The lower extremity of wound closing.

8th day. Emaciation of face marked, since yesterday; lint in the wound rapidly became offensive and required changing four or five times daily; patient takes a moderate quantity of nourishment.

10th day. She passed a bad night. The intestines became distended with gas, which caused more or less pain till three o'clock, A. M., when, after a copious alvine evacuation, she became comfortable, and slept till daylight. The patient looked badly, and had grown quite sallow and drowsy; moderate pain on pressure over the abdomen, significant of a low form of peritonitis; an opium suppository at noon. At 1.20 P. M. the patient suddenly began to sink; brandy was administered, but she did not rally, and died at 4.10 P. M.

*Remarks.* Should not every patient be tapped at some time previous to the time for the removal of the tumor, in order to add to the material to be considered in diagnosis? The information thus gained would be negative or otherwise. Take, for instance, a case like the one under consideration—an ovarian cystic colloid tumor, which, upon palpation, gave every indication of fluid contents. The rapidity of its growth, however, suggested the possibility of malignancy. Tapping would have revealed the fact that that which seemed to be fluid, was really colloid matter. Certainly the most practiced touch cannot discriminate between colloid matter and fluid, by palpation.

| | February | Operation | March | | | | | | Offensive odor about Patient. | Emaciation marked since yesterday. | | DEATH. |
|---|---|---|---|---|---|---|---|---|---|---|---|---|
| Date of Observation | 27 | 28 | 1 | 2 | 3 | 4 | 5 | 6 | 7 | 8 | 9 | 10 |
| From day of Operation | | 1 | 2 | 3 | 4 | 5 | 6 | 7 | 8 | 9 | 10 | 11 |

| | | | | | | | | | | | | | |
|---|---|---|---|---|---|---|---|---|---|---|---|---|---|
| Pulse | A.M. | 90 | 78 | 84 | 78 | 72 | 78 | 78 | 78 | 90 | 90 | 78 | 102 |
| | P.M. | 84 | 84 | 81 | 72 | 78 | 78 | 75 | 84 | 90 | 90 | 84 | 130* |
| Respiration | A.M. | 24 | 24 | 21 | 18 | 18 | 18 | 18 | 18 | 18 | 18 | 18 | 24 |
| | P.M. | 24 | 18 | 18 | 18 | 18 | 18 | 18 | 20 | 18 | 18 | 18 | |

Chart, showing Pulse, Respiration and Temperature.

This assemblage of signs—rapid growth, fluctuation upon palpation, and the negative results of tapping, are elements in the diagnosis of colloid cancer, or colloid metamorphosis of an ovarian cystic tumor.

Is any portion of the intestine ever adherent to the abdominal wall in the line of the incision? It is well known that, in the progressive growth of ovarian cysts, the intestines are pushed backwards and are so found at the time of operating. Mr. Christopher Heath, however, in the "New Sydenham Society's Biennial Retrospect of Medicine and Surgery, 1871 and '72," records a case in which he operated for ovarian disease, and in enlarging the abdominal wound with scissors, divided in three-quarters of its circumference an empty coil of intestine which was closely adherent to the abdominal wall. He stitched the intestine to the abdominal wound, and so formed an artificial anus. Mr. Heath remarks that he believes no similar case is on record. Should such an accident occur, he thinks the plan he adopted the best which can be carried out.

### SECTIO CADAVERIS, — HOURS AFTER DEATH.

By I. N. Danforth, M.D.

Body much emaciated ; some post-mortem discolorations posteriorly ; rigor mortis slight. The wound was quite firmly united, with the exception of the middle third, from which the pedicle protruded. From this portion of the wound a dark-brown or blackish, thin fluid escaped, in small quantity, which was afterwards found to proceed from the degenerating stump of the pedicle.

*Head.*—Not examined.

*Thorax.*—Neither the lungs, heart nor great vessels presented any pathological lesions. Considerable post-mortem hypostasis of blood was observed in the posterior portions of the lungs, and recent heart clots, small, soft, and dark red, were found in both ventricles.

*Abdomen.*—The peritoneum was mainly healthy ; near the margin of the wound, however, traces of recent inflammation were apparent. In two or three places the coils of intestines were glued together by recent but easily disrupted adhesions. The abdominal cavity contained a considerable quantity of straw-colored serum, in which floated a few—but *only* a few—fibrinous flakes. It was found that the pedicle of the tumor was isolated or pocketed by adhesions extending quite around it, and that it (the pedicle) was situated in the centre of this pocket, or cup-shaped depression. It was bathed in a dirty dark-brown, thin fluid, but this fluid was not very offensive. As this peculiar substance was not met with elsewhere, I infer that it was the product of the strangulated portion of the pedicle. Stomach, small intestines, liver, pancreas, spleen and kidneys, healthy. The large intestine was also in a healthy condition, except that anatomical and physiological puzzle, the "appendix vermiformis cæci," which was excessively distended by a deposit of colloid matter, precisely similar in gross and minute appearances to the matter composing the ovarian tumor, to the minute structure of which I shall allude more particularly hereafter.

The cavity of the uterus measured three and one-half inches in depth from the os to the fundus. The muscular wall of the uterus was considerably thickened, but otherwise healthy The right ovary was gone, but in its place the stump of the pedicle was found, which was apparently healing kindly. The left ovary consisted mainly of a bunch of cysts, from the size of a small shot up to that of a grape; each cyst was filled with colloid material precisely like that composing the right ovarian tumor; that is to say, the Graafian follicles of the left ovary had already undergone colloid degeneration, and had started on the same career which proved so disastrous to the right ovary.

Projecting from the anterior superior wall of the uterus just below the fundus, and apparently imbedded in its muscular structure, I found a growth about the shape and size of a goose's egg, which had the appearance of being a cyst, although to the touch it was exceedingly dense and hard—harder, indeed, than any cyst I ever saw. Upon attempting to incise this mass my knife immediately came upon something which seemed to be bone. A longitudinal incision was now carried through the entire extent of the outside portion or fibrous covering, which was carefully dissected off so as to perfectly expose the bony mass. It proved to be the remains of a uterine fibroid which had undergone quite complete calcareous degeneration. The microscopic structure of the colloid material of which this tumor was composed, proved to be unusually simple. If a thin specimen be held between the eye and a strong light, it appears to consist of a structureless, jelly-like material, traversed by a multitude of parallel white lines. If a section be examined by a low power (a one-inch objective) these lines appear to be made up of little granular bodies, arranged in regular rows, or files, but the intervening colloid material still appears hyaline and structureless; if the power be increased to 250 diameters, the rows of granules are seen to be rows of "cells"—that is, as we now understand the term. These cells possess neither "walls" nor nuclei; in fact, they are what would formerly have been called "free nuclei." Practically they are simply masses of germinal matter, engaged in the low-lived business of developing the so-called "colloid" material in which they are imbedded.

## Figures and Tables

**Figure f1:**